# Adenylate Cyclase 1 Links Calcium Signaling to CFTR-Dependent Cytosolic Chloride Elevations in Chick Amacrine Cells

**DOI:** 10.3389/fncel.2021.726605

**Published:** 2021-08-11

**Authors:** Li Zhong, Evanna L. Gleason

**Affiliations:** Department of Biological Sciences, Louisiana State University, Baton Rouge, LA, United States

**Keywords:** adenylate cyclase 1, cAMP, CFTR, nitric oxide, Ca^2+^, amacrine cell, GABAergic neuron

## Abstract

The strength and sign of synapses involving ionotropic GABA and glycine receptors are dependent upon the Cl^−^ gradient. We have shown that nitric oxide (NO) elicits the release of Cl^−^ from internal acidic stores in retinal amacrine cells (ACs); temporarily altering the Cl^−^ gradient and the strength or even sign of incoming GABAergic or glycinergic synapses. The underlying mechanism for this effect of NO requires the cystic fibrosis transmembrane regulator (CFTR) but the link between NO and CFTR activation has not been determined. Here, we test the hypothesis that NO-dependent Ca^2+^ elevations activate the Ca^2+^-dependent adenylate cyclase 1 (AdC1) leading to activation of protein kinase A (PKA) whose activity is known to open the CFTR channel. Using the reversal potential of GABA-gated currents to monitor cytosolic Cl^−^, we established the requirement for Ca^2+^ elevations. Inhibitors of AdC1 suppressed the NO-dependent increases in cytosolic Cl^−^ whereas inhibitors of other AdC subtypes were ineffective suggesting that AdC1 is involved. Inhibition of PKA also suppressed the action of NO. To address the sufficiency of this pathway in linking NO to elevations in cytosolic Cl^−^, GABA-gated currents were measured under internal and external zero Cl^−^ conditions to isolate the internal Cl^−^ store. Activators of the cAMP pathway were less effective than NO in producing GABA-gated currents. However, coupling the cAMP pathway activators with the release of Ca^2+^ from stores produced GABA-gated currents indistinguishable from those stimulated with NO. Together, these results demonstrate that cytosolic Ca^2+^ links NO to the activation of CFTR and the elevation of cytosolic Cl^−^.

## Introduction

Retinal amacrine cells (ACs) are interneurons located in the inner plexiform layer (IPL) where they make synaptic contacts with bipolar cells, ganglion cells, and other ACs. Most ACs are either GABAergic or glycinergic and affect excitatory synapses *via* presynaptic inhibition with output to bipolar cell synaptic terminals and postsynaptic inhibition onto ganglion cell dendrites and AC processes (MacNeil and Masland, 1998; Grimes, 2012; Diamond, 2017). Inhibition occurs when GABA or glycine binds to the ionotropic receptors which are ligand-gated Cl^−^ channels. The electrochemical gradient of Cl^−^ across the plasma membrane determines the postsynaptic voltage response because the membrane potential moves to the equilibrium potential for Cl^−^ when GABA- or glycine-gated channels are open. Because the equilibrium potential for Cl^−^ is largely determined by the cytosolic Cl^−^ concentration, regulation of cytosolic Cl^−^ is a key determinant of the strength and even sign of GABAergic and glycinergic synapses.

Cl^−^ cotransporter is a well-known mechanism that regulates cytosolic Cl^−^ concentration (Ben-Ari et al., 2012). The chloride cotransporters Na-K-2Cl (NKCC) and K-Cl (KCC) modulate the neuronal response to GABA and glycine by determining intracellular Cl^−^ level using the Na^+^ and K^+^ gradients, respectively (Russell, 2000; Delpire and Mount, 2002; Payne et al., 2003; Gamba, 2005). Previous work in our lab has shown that nitric oxide (NO) can stimulate the release of Cl^−^ from acidic intracellular stores in ACs by a mechanism involving the cystic fibrosis transmembrane conductance regulator (CFTR; Hoffpauir et al., 2006; Krishnan and Gleason, 2015; Krishnan et al., 2017).

CFTR is a Cl^−^ channel that has two membrane spanning domains (MSD), two cytosolic nucleotide binding domains (NBD), and a regulatory domain (RD) that contains numerous phosphorylation sites. It is encoded by the CFTR gene and mutations in this gene cause the deleterious effects observed in cystic fibrosis (Riordan et al., 1989; Tsui, 1992; Quinton, 1999). This disease is associated with reduced chloride permeability across the apical epithelial membrane (Quinton and Bijman, 1983; Widdicombe et al., 1985).

The chloride channel CFTR can be regulated by protein kinases and it is most strongly activated by protein kinase A (PKA; Picciotto et al., 1992; Gadsby and Nairn, 1999). CFTR can also be modulated by Ca^2+^ signaling as well as the cAMP pathway in epithelia (Kunzelmann and Mehta, 2013; Bozoky et al., 2017). Instead of stimulating the CFTR channel directly, Ca^2+^ can activate calcium-dependent adenylate cyclase (AdC) isoforms 1, 3, or 8, leading to the production of cAMP and activation of PKA. PKA can phosphorylate the CFTR RD and then activate the CFTR Cl^−^ channel (Halls and Cooper, 2011; Billet and Hanrahan, 2013). All AdC isoforms can be found in the central nervous system, but AdC8 is the least abundant (Xia et al., 1991; Mons et al., 1993). Although AdC3 is Ca^2+^/Calmodulin-activated, the sensitivity to Ca^2+^ is 100 times lower than that of AdC1 and activation requires the presence of other effectors such as forskolin or an activator of G proteins in human embryonic kidney 293 cells (Choi et al., 1992). The resting concentration of intracellular free Ca^2+^ in neurons typically ranges between 50 and 100 nM which matches the range of free Ca^2+^ producing half-maximal activation of AdC1 (Choi et al., 1992; Villacres et al., 1995; Masada et al., 2009).

We have previously demonstrated that NO leads to a CFTR-dependent release of internal Cl^−^ (Krishnan et al., 2017). However, the mechanism underlying this effect was unknown. In a previous study on the regulation of L-type Ca^2+^ channels in ACs, we learned that AdC1 is expressed in both cell bodies and cell processes of ACs in culture (Tekmen and Gleason, 2010). We have also reported that NO can increase cytosolic Ca^2+^ (Maddox and Gleason, 2017). Another possibility is that NO directly activates CFTR *via* s-nitrosylation. Analysis of the chicken CFTR sequence with the bioinformatics program RecSNO (Siraj et al., 2021) predicts nine likely s-nitrosylation sites. However, our observation of NO-dependent Ca^2+^ elevations leads us to prioritize investigating the role of AdC1. Hence, we hypothesized that the NO-generated Ca^2+^ elevations in ACs activate AdC1, produce elevations in cAMP, and ultimately phosphorylation and activation of CFTR *via* PKA.

To test this hypothesis, whole-cell voltage clamp recording was used to monitor changes in AC cytosolic Cl^−^ and Ca^2+^ imaging was used for monitoring cytosol Ca^2+^ concentration. We found that NO-dependent release of internal Cl^−^ was inhibited when either cytosolic Ca^2+^ was buffered by BAPTA or the cAMP pathway was suppressed by inhibitors. These data suggest that Ca^2+^ is crucial for NO-dependent release of Cl^−^ and it activates cAMP pathway through activation of AdC1.

## Materials and Methods

### Cell Culture

Retinas were dissected from 8-day chick embryos, and cells were plated on poly-L-Ornithine pre-processing dishes. Cells were first plated with Dulbecco’s modified Eagle’s medium (DMEM; Life Technologies), 5% fetal calf serum (Sigma, St. Louis, MO, USA), 100 U/ml penicillin, 100 μg/ml streptomycin, and 2 mM glutamine (Life Technologies), then fed every 2 days by replacing the media with feeding solution which contains neurobasal medium (Life Technologies), 1% B-27 nutrient medium and penicillin-streptomycin-glutamine (Life Technologies) for 2 weeks. For further details see Maddox and Gleason (2017). The experimental ACs were identified using established morphological criteria (Gleason et al., 1993) and were used for the experiment after 7–11 days in cultures.

### Electrophysiology

Culture dishes were anchored on the stage of an Olympus IX70 inverted microscope. An Ag-AgCl reference pellet electrode was submerged in 3M KCl solution and connected to the dish through a 1% agarose salt bridge. Patch pipettes were pulled from thick-walled borosilicate glass (1.5 mm outer diameter, 0.86 mm inner diameter) using a P-97 Flaming/Brown Puller (Sutter Instruments, Novato, CA, USA). The pipettes were chosen by a tip resistance between 8 and 12 MΩ. Whole-cell voltage clamp recordings were performed using Axopatch 1D amplifier, Digidata 1322A data-acquisition board, and Clampex 10.0 (Molecular Devices, Sunnyvale, CA, USA). The external solution was pushed to the dish with a constant flow rate 1 ml/min *via* a pressurized 8-channel perfusion system (Automate Scientific, Berkeley, CA, USA). Data were obtained from the ACs that were not in contact with other neurons. The current-voltage relationship was corrected for series resistance and junction potential errors (13 mV), calculated from pClamp 10.0 calculator. For voltage ramp experiments, the voltage was held at −70 mV for 150 ms, then stepped to −90 ms for 50 ms and ramped from −90 mV to +50 mV over 200 ms before stepping back to −70 mV. Each sweep contained two ramps, the first was for measuring the leak currents for subtraction from the second ramp recorded in the presence of GABA (20 μM). The total time of the protocol for each sweep was 2.7 s. The GABA_A_ receptor conductance was determined by calculating the slope of the current-voltage relationship from −60 mV to −40 mV.

For GABA pulse experiments, the voltage was held at −70 mV for 9.5 s, then exposed to GABA (20 μM) for 400 ms. Each sweep contained five GABA pulses and the total time of the sweep was 29 s. The largest GABA pulse response was chosen for analysis. The GABA-gated current amplitude with treatment was calculated by subtracting the control GABA-gated current with no test treatment. Each replicate indicates an individual cell. The experiments in Figure 5 (NO vs. cAMPct) and in Figure 8D (NO vs. cAMPct + ionomycin) were analyzed with the same NO control group because the experiments were done on the same group of cells. The external solution and GABA containing external solution flowed into the test dish through fast-step perfusion system SF-77C *via* 3-barrel square glass (Warner Instruments, Hamden, CT, USA). All experiments were performed at room temperature.

**Figure 1:**
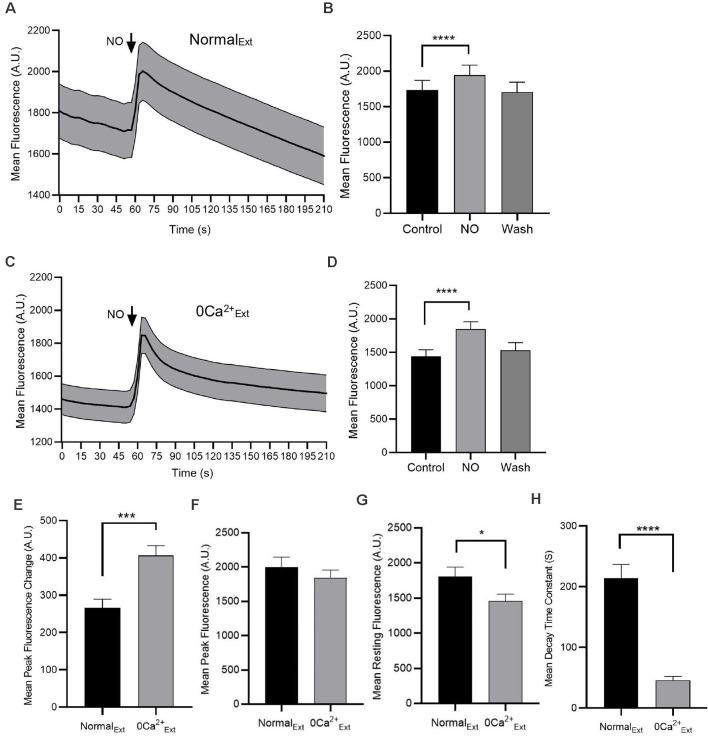
Nitric oxide (NO) increases cytosolic Ca^2+^ concentration. Fluorescence intensity measurements from Amacrine cells (ACs) loaded with Oregon Green BAPTA-1 488-AM are shown **(A,C)**. **(A)** In the normal external solution, NO produces an increase in cytosolic Ca^2+^. **(B)** The quantified mean fluorescence ± SEM; *n* = 45. For the control group, the mean value is calculated as the average of the last 30 s data before adding NO. For the NO group, the mean value is calculated as the average of the middle 30 s during the NO response. For the wash group, the mean is calculated by using the average of the data from 150 to 177 s. ^****^P < 0.0001 (paired *t*-test). **(C)** In the absence of Ca^2+^ (0 Ca^2+^_Ext_), NO increases cytosolic Ca^2+^. **(D)** The quantified mean fluorescence ± SEM; *n* = 54. ^****^P < 0.0001 (paired *t*-test). **(E)** The quantified mean peak fluorescence ± SEM after subtracting baseline in normal external condition, *n* = 45 and in zero Ca^2+^ external condition, *n* = 54. ***P < 0.001 (unpaired *t*-test).** (F)** The quantified mean peak fluorescence ± SEM in normal external condition, *n* = 45 and in zero Ca^2+^ external condition, *n* = 54. *P* = 0.38 (unpaired *t*-test). **(G)** The quantified mean resting fluorescence ± SEM in normal external condition, *n* = 45 and in zero Ca^2+^ external condition, *n* = 54. *P < 0.05 (unpaired *t*-test). **(H)** The quantified mean decay time constant ± SEM in normal external condition, *n* = 33 and in zero Ca^2+^ external condition, *n* = 49. Data for each cell are fitted with one phase decay exponential curve. ****P < 0.0001 (unpaired *t*-test).

**Figure 2:**
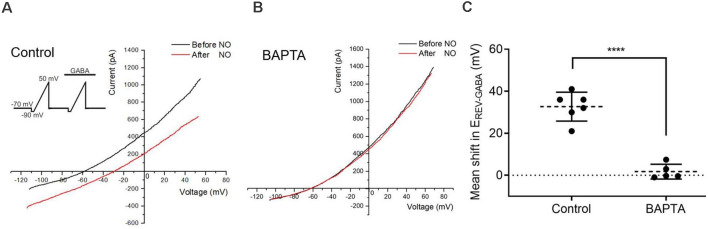
Calcium elevations are required for the NO-dependent release of internal Cl^−^. **(A,B)** The current-voltage relationship for the GABA-gated current (20 μM) before and after NO. Inset shows the voltage ramp protocol with GABA delivered during the second ramp. Under control conditions, E_REV-GABA_ shifts to the right. With the Ca^2+^ chelator, BAPTA (50 μM) in the recording pipet, NO fails to elicit the E_REV-GABA_ shift. **(C)** The quantified mean shift in E_REV-GABA_ ± SEM; control, *n* = 6; BAPTA, *n* = 5. ****P < 0.0001 (unpaired *t*-test).

**Figure 3:**
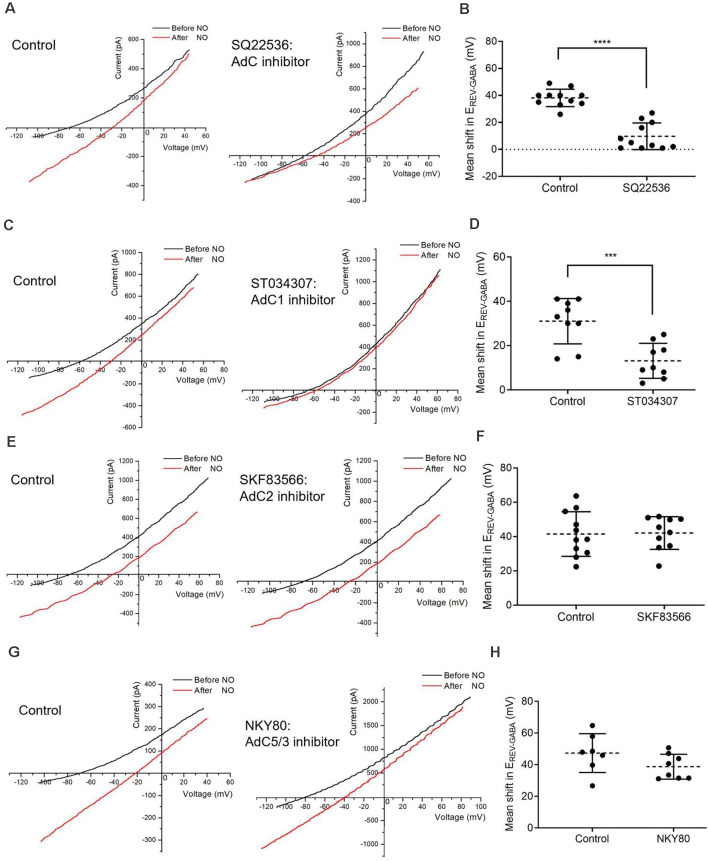
AdC1 inhibitors suppress NO-dependent Cl^−^ release. **(A)** Data from cells recorded under control condition (left) or pre-incubated with general AdC inhibitor, SQ22536 (100 nM) for 30 mins (right) showing reduced shift amplitudes. **(B)** The quantified mean shift in E_REV-GABA_ ± SEM; *n* = 11 each. ****P < 0.0001 (unpaired *t*-test). **(C)** Pre-incubation with AdC 1 inhibitor, ST034307 (100 nM) for 30 mins, suppresses the NO-dependent shift in E_REV-GABA_. **(D)** The quantified mean shift in E_REV-GABA_ ± SEM; *n* = 9. ***P < 0.001 (unpaired *t*-test). **(E)** Pre-incubation with the AdC 2 inhibitor, SKF83566 (10 μM) for 20 mins, does not inhibit the NO-dependent shift in E_REV-GABA_. **(F)** The quantified mean shift in E_REV-GABA_ ± SEM; control, *n* = 11; SKF83566, *n* = 10. *P* = 0.91 (unpaired *t*-test). **(G)** Pre-incubation with the AdC 5/3 inhibitor, NKY80 (200 μM) for 20 mins does not block the NO-dependent shift. **(H)** The quantified mean shift in E_REV-GABA_ ± SEM; control, *n* = 7; NKY80, *n* = 8. *P* = 0.13 (unpaired *t*-test).

**Figure 4:**
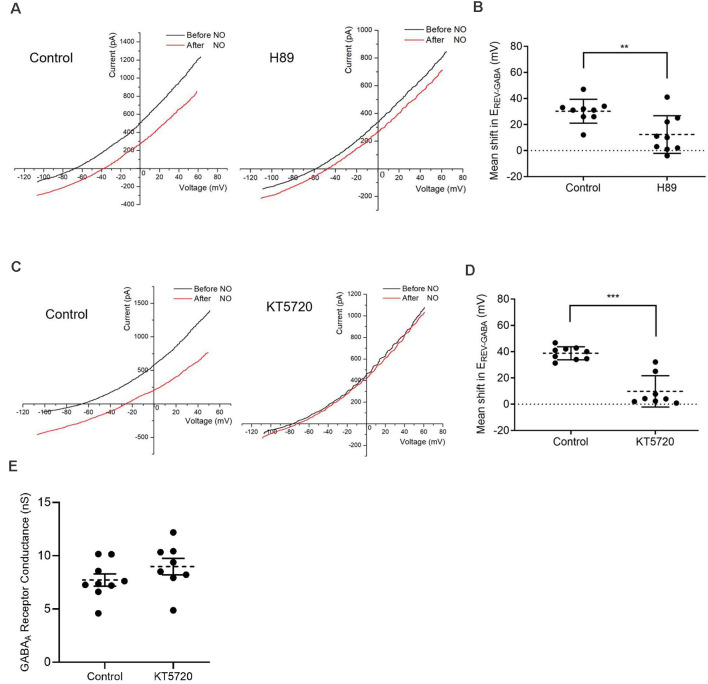
Protein kinase A (PKA) activity is required for the NO-dependent shift in E_REV-GABA_. **(A)** Date from cells recorded under control condition (left) or pre-incubated with a kinase inhibitor, H89 (1 μM) for 30 min (right) that suppresses the NO-dependent shift in E_REV-GABA_. **(B)** The quantified mean shift in E_REV-GABA_ ± SEM; *n* = 9. **P < 0.01 (unpaired *t*-test). **(C)** Pre-incubation with selective PKA inhibitor, KT5720 (300 nM, 20 min), also reduces E_REV-GABA._ **(D)** The quantified mean shift E_REV-GABA_ ± SEM; control, *n* = 9; KT5720, *n* = 8. ***P < 0.001 (Welch’s unpaired *t*-test). **(E)** Quantified mean GABA_A_ receptor conductance ± SEM; control, *n* = 9; KT5720, *n* = 8. *P* = 0.2057 (unpaired *t*-test).

**Figure 5:**
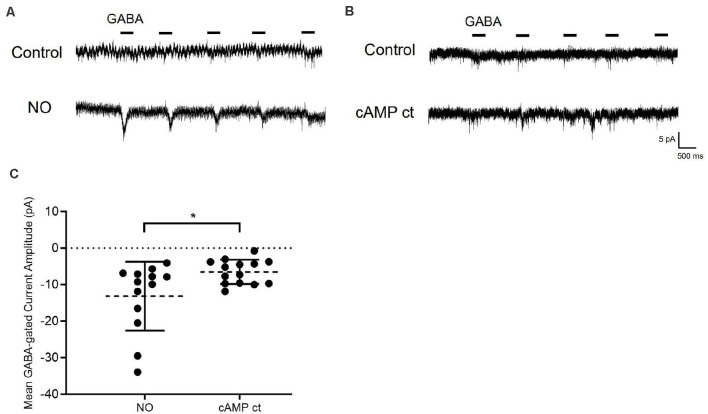
Elevating cAMP in the absence of NO promotes Cl^−^ release from the internal store. **(A)** Sample traces from cells are held at −70 mV with GABA (20 μM) applied for 400 ms. In the presence of NO, a GABA-gated inward current appears indicating that Cl^−^ had been released into the cytosol. **(B)** In different cells, exposure to a cocktail of reagents selected to elevate cAMP (cAMP ct: forskolin, 1 μM; 8-bromo-cAMP, 100 μM; IBMX, 20 μM) but no NO, results in a small GABA-dependent inward current. **(C)** The quantified mean GABA-gated current amplitude ± SEM; NO, *n* = 13; cocktail, *n* = 14. *P < 0.05 (Welch’s unpaired *t*-test).

**Figure 6:**
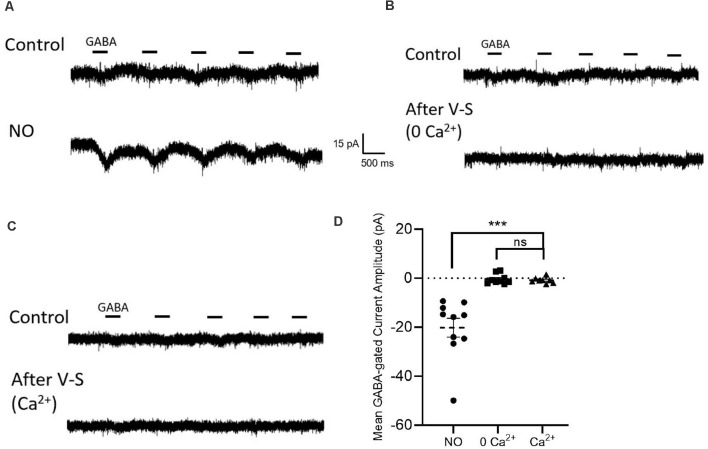
Influx of external Ca^2+^ fails to release Cl^−^ from the internal store.** (A)** Sample traces from cells are held at −70 mV with GABA (20 μM) applied for 400 ms. With NO stimulation, GABA-gated inward currents generate. **(B)** Voltage-step control settings in zero Ca^2+^ zero Cl^−^ external environment. Sample traces from cells that the top trace is stimulated with GABA pulse protocol, the bottom trace is repeated with GABA pulse protocol after voltage step stimulation. **(C)** Same experiment setting in Ca^2+^ (3 mM) external solution. **(D)** Quantified mean GABA-gated current amplitude ± SEM; NO control, *n* = 10; 0 Ca^2+^ control, *n* = 9; Ca^2+^, *n* = 8; *p* = 0.6; ns: not significant. ***P < 0.001 (repeated-measures ANOVA).

**Figure 7:**
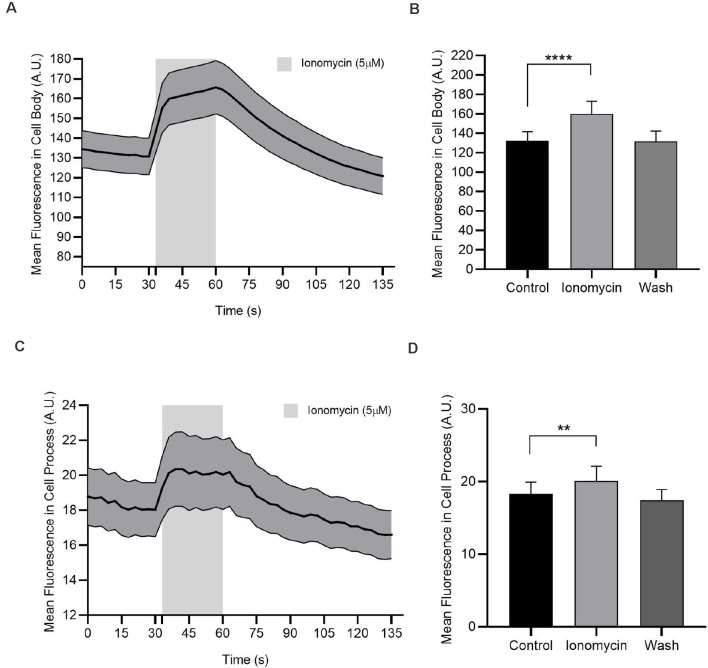
In the absence of extracellular Ca^2+^, ionomycin releases Ca^2+^ from internal stores. Calcium imaging experiments of AC cell body and cell process are loaded with Oregon Green BAPTA-1 488-AM. **(A)** Ionomycin (5 μM) increases cytosolic Ca^2+^ in the cell body. **(B)** Quantified mean fluorescence ± SEM; *n* = 73. For the control and ionomycin groups, mean value is calculated as the average of all test time in a condition. For the wash, the mean is calculated from the time point 93–120 s. ****P < 0.0001 (paired *t*-test). **(C)** Ionomycin (5 μM) increases cytosolic Ca^2+^ in the cell process. **(D)** Quantified mean fluorescence ± SEM; *n* = 58. Mean values are calculated as in **(C)**. **P < 0.01 (paired *t*-test).

**Figure 8:**
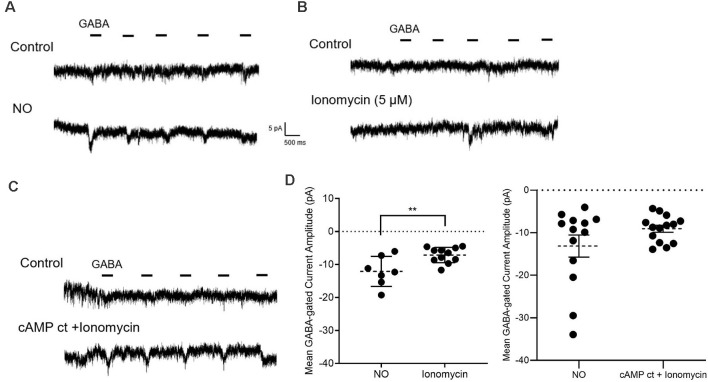
Pairing elevated cAMP and Ca^2+^ mimics the effects of NO.** (A–C)** Sample traces from GABA pulse recording (20 μM) in zero external Ca^2+^ and zero Cl^−^ internal and external solutions. The GABA-dependent inward current is elicited by NO (50 μl) in **(A)** by Ca^2+^ ionophore, ionomycin (5 μM) in (**B)**, and by co-application of cAMP ct and ionomycin (5 μM) in **(C). (D)** Quantified mean GABA-gated current amplitude ± SEM; NO, *n* = 7; ionomycin, *n* = 11. NO, *n* = 13 (control cells are replotted from Figure 5D; see “ Materials and Methods” section); cocktail and ionomycin, *n* = 14. **P < 0.01 (left: unpaired *t*-test, right: Welch’s unpaired *t*-test).

### Solutions

All components were purchased from Sigma-Aldrich (St. Louis, MO, USA) unless otherwise indicated. The normal external solution contained (in mM): NaCl (116.7), KCl (5.3), tetrathylammonium (TEA)-Cl (20.0), CaCl_2_ (3), MgCl_2_ (0.41), glucose (5.6), HEPES (10.0), pH adjusted to 7.4 with NaOH. For voltage ramp recordings, Tetrodotoxin citrate (TTX; 300 nM, Abcam, Cambridge, UK) and La^3+^ (50 μM) were added to block voltage-gated Na^+^ and Ca^2+^ currents, respectively. The normal internal solution contained (in mM): Cs-acetate (100.0), CsCl (10.0), CaCl_2_ (0.1), MgCl_2_ (2.0), HEPES (10.0), and EGTA (1.0), pH adjusted to 7.4 with CsOH. For GABA pulse experiments, zero Cl^−^ external solution was made with (in mM): Na-methanesulfonate (145.0), glucose (5.6), and 10.0 HEPES, pH adjusted to 7.4 with NaOH. The zero Cl^−^ internal solution was made with (in mM): Cs-methanesulfonate (125.0) and HEPES (10.0), pH adjusted to 7.4 with CsOH. The agarose salt bridge was placed close to the outflow in order to minimize Cl^−^ leaking onto ACs during recording. For Ca^2+^ imaging experiments, zero Ca^2+^ external solution contained (in mM): NaCl (141.5), KCl (5.37), MgCl2 (0.41), glucose (5.6), HEPES (3.02), pH adjusted to 7.4 with NaOH. For all electrophysiological experiments, the internal solution was supplemented with an ATP regeneration system containing creatine phosphokinase (50 U/ml), creatine phosphate (20 mM), ATP disodium (1 mM), ATP dipotassium (3 mM), and GTP disodium (2 mM). The NO-saturated solution was prepared by bubbling Fisher ultra-distilled pure water with argon for 15 min, then bubbled with pure NO gas for 15 min. NO solution was delivered by injection into the external solution perfusion line. The concentration of NO at the perfusion outlet was in the 100’s nM-low μM range. NO (30–50 μl) has been estimated to reach the recorded cell in ~3 s and to remain for ~3–5 s (Hoffpauir et al., 2006).

### Reagents

All reagents were purchased from Tocris (Bristol, UK). The 1,2-Bis(2-aminophenoxy)ethane-N,N,N′,N′-tetraacetic acid tetrakis (acetoxymethyl ester; BAPTA, 50 μM) was prepared as a 50 mM stock in DMSO and diluted in internal solution. The AdC inhibitor (SQ 22536, 100 nM), AdC 1 inhibitor (ST 034307, 100 nM), AdC2 inhibitor (SKF 83566 hydrobromide, 10 μM), AdC 5/3 inhibitor 2-Amino-7-(2-furanyl)-7,8-dihydro-5(6*H*)-quinazolinone (NKY 80, 200 μM), PKA inhibitor *N*-[2-[[3-(4-Bromophenyl)-2-propenyl]amino]ethyl]-5-isoquinolinesulfonamide dihydro­chloride (H89 dihydrochloride, 1 μM) and selective PKA inhibitor (KT 5720, 300 nM) were prepared as 1,000X stocks in DMSO and diluted to 1X in the external solution. Cells were preincubated with inhibitors at 37°C in 5% CO_2_ for 20–30 mins before recording. The Cell-permeable cAMP analog (8-Bromo-cAMP, sodium salt, 100 μM), the phosphodiesterase inhibitor (non-selective; 3-Isobutyl-1-methylxanthine, 20 μM), and AdC activator forskolin (1 μM) were prepared as 1,000X stocks in DMSO and diluted to 1X in the external solution. 8-Bromo-cAMP, IBMX, and forskolin were applied during recording *via* the perfusion. The calcium ionophore (ionomycin, 5 μM) was prepared as a 5 mM stock in DMSO and diluted in the external solution.

### Calcium Imaging

The cell-permeable fluorescence Ca^2+^ indicator Oregon Green 488 Bapta-1, AM (OGB, 2 μM, Life Technologies, Grand Island, NY) stock solution (2 mM) was mixed with Pluronic F-127 (2.5% w/v in DMSO, Life Technologies) 1:1 sonicated, then 2 μl was added to 1 ml of Hank’s buffered saline solution (HBSS), vortexed and sonicated for 30 s to produce the loading solution. The culture media was replaced with OGB in HBSS and cells were incubated for 1 h at room temperature. For zero Ca^2+^ experiments, the perfusion was switched to zero Ca^2+^ external solution prior to the start of the recording. Images were taken every 3 s with 1 s exposure time on an inverted Olympus IX70 microscope (Center Valley, PA) fitted with a SensiCam QE (Cooke, Kelheim, Germany). Data were collected from regions of interest (ROIs) in ACs bodies and cell processes. For analysis of the effect of NO, the mean value of the control group was calculated by taking the average of the last 30 s before NO application, the mean value of the NO group was calculated by taking the average of the middle 30 s during NO application. The average of data points from 150 s to 177 s was the mean value for the wash group. The mean peak fluorescence change was calculated by subtracting the mean of baseline fluorescence intensity (30 s before NO response) from the peak NO response for data from each ROI. For analysis of the effect of ionomycin, the means of all data points collected before, and during ionomycin application were calculated. Wash data were calculated from a window of time from 93 s to 120 s. Images acquisition and analysis were achieved using Slidebook 5.0 software.

### Data Analysis

Analysis of voltage ramps experiments, the current and voltage relationships were calculated and plotted in Origin 8.0 (OriginLab, Northampton, MA, USA). Clampfit 10.0 software was used to exhibit the sweep traces and analyze the currents. Analysis of GABA pulse experiments, GABA-gated inward currents were displayed in Clampfit 10.0 and filtered with Gaussian low pass at 300 Hz. Data were calculated and plotted in Origin 8.0. Calcium imaging data were analyzed in Slidebook 5.0. All the descriptive statistics, unpaired *t*-tests, paired *t*-tests, and Welch’s *t*-tests and ANOVA were performed on Prism 8 (GraphPad Software, La Jolla, CA, USA). Data were reported as means ± SEM. The *P*-value of significance was <0.05.

## Results

### NO Releases Ca^2+^ From Stores

We have previously shown that the NO donor SNAP can enhance intracellular Ca^2+^ in ACs *via* activation of transient receptor potential canonical channel 5 (TRPC5; Maddox and Gleason, 2017; Maddox et al., 2018). To confirm the NO-bubbled external solution also elevates Ca^2+^ levels, cells were loaded with Oregon Green BAPTA-1 488-AM (OGB, 2 μM) and fluorescence intensity was monitored over time. With application of NO in normal external solution, the intracellular Ca^2+^ increased by about 12% (control, 1,736 ± 133.4 AU; NO, 1943 ± 140.5 AU; *n* = 45; *p* < 0.0001, paired *t*-test, Figures 1A,B). To determine whether this NO exposure could elicit the release of Ca^2+^ from stores, Ca^2+^ was removed from the external solution. NO elicited a 28% cytosolic Ca^2+^ elevation in the absence of extracellular Ca^2+^ (control, 1,441 ± 97.1 AU; NO, 1848 ± 109 AU; *n* = 54; *p* < 0.0001, paired *t*-test, Figures 1C,D). After subtracted baseline fluorescence, cells in zero Ca^2+^ extracellular condition had higher cytosolic Ca^2+^ increase than that in normal external condition (normal external, 266.1 ± 23.2 AU; *n* = 45; zero Ca^2+^ external, 406.8 ± 26.1 AU; *n* = 54; *p* < 0.001, unpaired *t*-test, Figure 1E). However, the maximum NO responses in normal and zero Ca^2+^ external were not statistically different (normal external, 2,002 ± 141.2 AU; *n* = 45; zero Ca^2+^ external, 1,848 ± 109 AU; *n* = 54; *p* = 0.38, unpaired *t*-test, Figure 1F). The higher magnitude of change NO-dependent cytosolic Ca^2+^ in zero Ca^2+^ external is due to the lower resting cytosolic Ca^2+^ level (normal external, 1,808 ± 131.7 AU; *n* = 45; zero Ca^2+^ external, 1,460 ± 94.2 AU; *n* = 54; *p* < 0.05, unpaired *t*-test, Figure 1G). The decay time constant in normal external was larger than that in zero Ca^2+^ external condition, suggesting that the later part of the response was more dependent upon extracellular Ca^2+^, consistent with activation of TRPC5 (Maddox et al., 2018; normal external, 213.8 ± 22.9, *n* = 33 s; zero Ca^2+^ external, 45.8 ± 6.2 s, *n* = 49; *p* < 0.0001, unpaired *t*-test, Figure 1H). Together, these results suggest that NO-bubbled solutions can elicit both Ca^2+^ influx across the plasma membrane and release of Ca^2+^ from internal stores.

### Ca^2+^ Elevations Are Required for the NO-Dependent Release of Internal Cl^−^

To determine whether the elevation in cytosolic Ca^2+^ is required for the release of internal Cl^−^, the effect of BAPTA was tested. To estimate cytosolic Cl^−^ concentration, the NO-dependent shift in the reversal potential of GABA-gated current (E_REV-GABA_) was determined by delivering voltage ramps during the application of GABA to activate GABA_A_ receptors. As previously demonstrated with gramicidin perforated-patch recordings (Hoffpauir et al., 2006), the resting intracellular Cl^−^ level is relatively low in ACs and similar to our pipet solution Cl^−^ concentration. E_REV-GABA_ was determined before and after NO to track cytosolic Cl^−^ concentration changes. Under control conditions, the reversal potential had a positive shift with NO, indicating an increase in cytosolic Cl^−^ (control, 32.7 ± 2.8 mV, *n* = 6, Figure 2A). The Ca^2+^ chelator BAPTA (50 μM) was applied *via* the recording pipette. In cells loaded with BAPTA, the NO-dependent reversal potential shift was strongly inhibited (BAPTA, 1.7 ± 1.6 mV, *P* < 0.0001, *n* = 5, Figures 2B,C). These results indicated that calcium elevations are required for the NO-dependent release of internal Cl^−^.

### Only AdC1 Inhibitors Suppress the NO-Dependent Release of Cl^−^

To investigate the involvement of AdC1 in release of Cl^−^ from the internal store, the effects of AdC inhibitors were examined. SQ22536 (100 nM), a general AdC inhibitor, suppressed the shift in E_REV-GABA_ (control shift, 38.2 ± 1.9 mV; SQ22536 shift, 9.7 ± 3.0 mV; *n* = 11 for both groups; *p* < 0.0001, unpaired *t*-test, Figures 3A,B). The AdC1 inhibitor, ST034307 (100 nM) was also effective; providing evidence for the involvement of AdC1, specifically (control shift, 31.0 ± 3.4 mV; ST 034307 shift, 13.1 ± 2.6 mV; *n* = 9 each; *p* < 0.001, unpaired *t*-test, Figures 3C,D). Neither the AdC2 inhibitor SKF83566 (10 μM) nor the AdC3 and 5 inhibitor (NKY 80, 200 μM) inhibited the NO-dependent shift in E_REV-GABA_ (control shift, 41.5 ± 3.9 mV, *n* = 11; SKF83566 shift, 42.1 ± 3.0 mV, *n* = 10; *p* = 0.9079; control shift, 47.3 ± 4.6 mV, *n* = 7; NKY 80 shift, 38.7 ± 2.8, *n* = 8; *p* = 0.1254; unpaired *t*-tests, Figures 3E–H). Together, these results suggest that AdC1, specifically, is involved in the NO-dependent internal Cl^−^ release.

### PKA Inhibitors Disrupt the NO-Dependent Release of Internal Cl^−^

To examine the role of PKA in determining cytosolic Cl^−^ concentration, two PKA inhibitors (H89 1 μM, KT5720 300 nM) were employed. Both inhibitors were effective in suppressing the NO-dependent internal Cl^−^ release (control shift, 30.2 ± 3.1 mV; H89 shift, 12.3 ± 4.8 mV, preincubated for 30 min; *n* = 9 for both groups; *p* < 0.01, unpaired *t*-test, Figures 4A,B; control shift, 38.8 ± 1.7 mV, *n* = 9; KT5720 shift, 9.8 ± 4.2 mV, preincubated for 20 min, *n* = 8; *p* < 0.0001, Welch’s unpaired *t*-test, Figures 4C,D). In addition, there is evidence that PKA directly modulates GABA_A_ receptors in multiple cell types including retinal neurons (Porter et al., 1990; Wong and Moss, 1992; Feigenspan and Bormann, 1994; Veruki and Yeh, 1994; Wexler et al., 1998). To test this possibility that inhibition of PKA might affect the GABA_A_ receptor itself by altering mean open time or receptor sensitivity, we compared the conductance of the GABA-gated current in control and KT5720 pretreatment conditions. No effects of PKA inhibition on the conductance were observed (control, 7.72 ± 0.58 nS, *n* = 9; KT5720, 8.98 ± 0.77 nS, *n* = 8; *p* = 0.2057, unpaired *t*-tests, Figure 4E). In sum, these results indicate a role for AdC1-dependent signaling *via* cAMP and PKA in the activation of CFTR.

### Elevating cAMP in the Absence of NO Promotes Cl^−^ Release From the Internal Store

If AdC1 and PKA are sufficient for the release of Cl^−^ from internal stores, we should be able to bypass NO and directly activate the cAMP pathway to release Cl^−^ into the cytosol. To achieve this, we used a combination of reagents to create a cAMP “cocktail” (cAMP ct) composed of the AdC activator, forskolin (1 μM); the cAMP analog, 8-bromo-cAMP (100 μM), and the phosphodiesterase inhibitor, IBMX (20 μM). To maximize our ability to detect Cl^−^ release and to ensure that we were measuring Cl^−^ originating from the internal store, we employed an alternative experimental strategy. GABA-gated currents were recorded in Cl^−^-free internal and external solutions. Because E_REV-GABA_ is not measurable under these conditions, cells were held at −70 mV and GABA (20 mM) was applied for 400 ms (five times /sweep). GABA-gated inward currents were initially very small then disappeared completely as the Cl^−^-free pipet solution washed out residual Cl^−^ from the cell. After NO, however, GABA-gated inward currents were observed indicating that sequestered Cl^−^ had been released into the cytosol and then exited *via* open GABA_A_ receptors on the plasma membrane (Figure 5A).

The cAMP pathway inhibitor experiments suggest that activation of the cAMP pathway is necessary for the NO-dependent shift in E_REV-GABA_. To test whether cAMP pathway activity is sufficient, cells were exposed to cAMP ct designed to elevate cAMP levels and PKA activity. In the GABA pulse experiment, the cAMP ct generated a small inward GABA-gated current (Figure 5B). Although the GABA responses were elicited by cAMP ct, they were significantly smaller than those recorded in response to NO (Figure 5C). These results indicate that elevating cAMP signaling alone has a small effect on releasing Cl^−^ from internal store (NO, −13.1 ± 2.6 pA, *n* = 13; cAMP ct −6.5 ± 0.9 pA, *n* = 14; *p* < 0.05, Welch’s unpaired *t*-test).

### Activation of Voltage-Gated Ca^2+^ Channels Does Not Elicit Cl^−^ Release

The activity of AdC1 relies on Ca^2+^. To determine whether a Ca^2+^ elevation alone is sufficient to activate the pathway and cause the release of internal Cl, a series of 10 sweeps, 100 ms voltage steps from −70 mV to 0 mV of each sweep were delivered after the GABA pulse control sweeps (Figures 6B,C). For these experiments, 3 mM Ca^2+^ was added to the external solution (Figure 6C). After the voltage steps, subsequent GABA pulses did not elicit inward currents (NO control, −20.2 ± 3.8 pA, *n* = 10; 0Ca^2+^ control, −0.4 ± 0.7 pA, *n* = 9; Ca^2+^, −0.8 ± 0.4 pA, *n* = 8; *p* = 0.6, repeated-measures ANOVA, Figures 6A–D). These results suggest that Ca^2+^ influx *via* voltage-gated Ca^2+^ channels is not effective in activating efflux of Cl^−^ from internal stores.

### Ionomycin Releases Ca^2+^ From Internal Stores in the Absence of Extracellular Ca^2+^

If the influx of Ca^2+^ through voltage-gated Ca^2+^ channels is ineffective in elevating cytosolic Cl^−^ but Ca^2+^ elevations are required, perhaps it is Ca^2+^ released from internal stores specifically, that links NO to Cl^−^ efflux. To test this, we first confirmed that we could use ionomycin in Ca^2+^-free external solution to elicit the release from Ca^2+^ stores. OGB-loaded ACs were monitored during exposure to ionomycin (5 μM). Data were captured in AC cell bodies and cell processes. With ionomycin, intracellular Ca^2+^ increased by about 21% and 9.8% in cell bodies and processes, respectively (cell bodies: control, 132.3 ± 9.3 AU; ionomycin 160 ± 13 AU; *n* = 73; *p* < 0.0001, paired *t*-test, Figures 7A,B; cell processes: control, 18.3 ± 1.6 AU; ionomycin 20.1 ± 2.0 AU; *n* = 58; *p* < 0.01, paired *t*-test, Figures 7C,D). These data show that ionomycin is effective in releasing store Ca^2+^ in ACs.

### Pairing Elevated cAMP and Ca^2+^ Release Mimics the Effects of NO

To test whether the release of store Ca^2+^ can stimulate Cl^−^ release from the Cl^−^ internal store, GABA pulse data were gathered before and after exposure to ionomycin. These experiments were done in the absence of internal and external Cl^−^, and external Ca^2+^. With the addition of ionomycin (5 μM), small GABA-gated inward currents were elicited but were significantly smaller than those elicited by NO (NO, −12.1 ± 1.7 pA, *n* = 7; ionomycin, −7.1 ± 0.7 pA, *n* = 11; *p* < 0.01, unpaired *t*-test, Figures 8A,B,D). Either store release of Ca^2+^ or elevation of cAMP was less effective than NO in eliciting release of internal Cl^−^ (Figures 5B,C, 8B,D). As such, we hypothesized that releasing stored Ca^2+^ and the downstream effects of elevating cytosolic cAMP would have synergistic effects on Cl^−^ release. To test this, ionomycin and cAMP ct were co-applied and the effect on GABA-gated currents in zero Cl^−^ and zero Ca^2+^ external solutions was determined under these conditions, the effects of NO could be mimicked (NO, −13.1 ± 2.6 pA, *n* = 13; cAMP ct + ionomycin, −9.0 ± 0.8 pA, *n* = 14; *p* = 0.1588, Welch’s unpaired *t*-test, Figures 8C,D). Together, these results provide evidence that Ca^2+^-dependent AdC1 provides a link between NO-dependent cytosolic Ca^2+^ signaling and cytosolic Cl^−^ concentration.

## Discussion

Here we show that by eliciting Ca^2+^ release, NO sets the conditions for CFTR-dependent efflux of Cl^−^ from internal stores. We establish that Ca^2+^ store release, AdC1 activation, and PKA activation are all necessary for the NO-dependent release of Cl^−^ into the cytosol. Our attempts to bypass NO and the Ca^2+^ elevations by experimentally elevating cytosolic cAMP engendered Cl^−^ release, but these manipulations were not as effective as NO itself. Combining cAMP elevations with the release of stored Ca^2+^ generated Cl^−^ release indistinguishable from NO. This implies that there might be an additional Ca^2+^-dependent component to this mechanism that is as yet, unidentified. Taken together, these results establish that AdC1 links calcium signaling and the cAMP pathway to the regulation of cytosolic Cl^−^ *via* CFTR.

### CFTR and cAMP Signaling Interactions

It is well established that activation of CFTR is modulated by the cAMP signaling pathway (Anderson and Welsh, 1991). Here we report that AdC1, specifically, is a site of interaction between Ca^2+^ and cAMP for CFTR regulation in chick ACs. Functional coupling between AdC1 and CFTR has been demonstrated in human bronchial cell lines (Namkung et al., 2010; Lérias et al., 2018) airway, and intestinal epithelia (Benedetto et al., 2017) and human fetal lung explants (Brennan et al., 2016). Here, we demonstrate for the first time, a similar relationship in neurons.

A common theme for CFTR is that it forms function partnerships with other transport proteins (Li and Naren, 2010). It has become well established in epithelial cells that a physical and functional relationship exists between CFTR and TMEM16A (anoctamin 1), a Ca^2+^-activated Cl^−^ channel (Ousingsawat et al., 2011; Benedetto et al., 2017, 2019). Interestingly, TMEM16A has been localized to AC-AC GABAergic synapses in the mouse retina (Jeon et al., 2013) and we have evidence that it is expressed in cultured GABAergic chick ACs at the mRNA and protein levels (unpublished observations). Two emerging properties of TMEM16A make it a good candidate for an additional Ca^2+^-dependent component of the CFTR activation mechanism in ACs. First, in epithelia, the co-expression of TMEM16A and CFTR confers a system where cytosolic Ca^2+^ elevations and activation of the cAMP pathway become intertwined by the activity of Ca^2+^-dependent AdCs, mostly likely AdC1 (Benedetto et al., 2017; Lérias et al., 2018). Furthermore, although the mechanism is not understood, expression of TMEM16A enhances G-protein coupled receptor-mediated Ca^2+^ release *via* the inositol 1,4,5-trisphosphate receptors (IP_3_R; Schreiber et al., 2015; Benedetto et al., 2017). It may be that TMEM16A serves a similar function in ACs, even in the absence of receptor activation. Second, it has been elegantly demonstrated that TMEM16A is activated by Ca^2+^ released from stores exclusively, in epithelia (Cabrita et al., 2017) and sensory neurons (Jin et al., 2013). These reports align with our observation that only store-released Ca^2+^ is effective in eliciting elevations in cytosolic Cl^−^.

### Ca^2+^ Signaling for Cl^−^ Regulation

We find that Ca^2+^ influx is not effective in enhancing cytosolic Cl^−^ but that Ca^2+^ store release is. AdC1 is typically expressed on the plasma membrane as are voltage-gated Ca^2+^ channels, so our observations suggest that the expression of these two membrane proteins is significantly non-overlapping. Furthermore, the effectiveness of Ca^2+^ store release raises the possibility that the Ca^2+^ store release mechanism is localized together with Cl^−^ release sites.

The mechanism by which NO releases internal Ca^2+^ in ACs remains unknown. We have previously determined that the effect of NO on cytosolic Cl^−^ in ACs is independent of soluble guanylyl cyclase activity (Hoffpauir et al., 2006). This observation raises the possibility that NO functions *via* S-nitrosylation. NO can activate ryanodine receptors (RyRs) *via* S-nitrosylation (Eu et al., 2000), and NO-dependent release of store Ca^2+^ *via* RyRs was shown to affect cerebellar neuron synaptic plasticity as well as cell death (Kakizawa et al., 2012). In addition, the IP_3_R can be S-nitrosylated and elicit Ca^2+^ release from the IP_3_-sensitive store in human neutrophils (Pan et al., 2008). The interplay between IP_3_R and RyR has been demonstrated in the production of cytosolic Ca^2+^ waves (Boittin et al., 1999; Gordienko and Bolton, 2002), and both receptors are expressed in cultured ACs (Warrier et al., 2005; Sen et al., 2007).

Acidic organelles such as lysosomes (Patel and Docampo, 2010) are another potential source of Ca^2+^. This Ca^2+^ store can be mobilized in response to nicotic acid adenine dinucleotide phosphate (NAADP) activation of Ca^2+^ permeable two-pore channels (TPCs; Calcraft et al., 2009). However, the only link between NO and the pathway we have identified is that cAMP can regulate NAADP production (Wilson and Galione, 1998). Nonetheless, it is an intriguing possibility that acidic organelles might function in both mobilizing Ca^2+^ and regulating Cl^−^ levels in the cytosol.

Can signaling molecules other than NO link cytosolic Ca^2+^ and cytosolic Cl^−^? Chick ACs have been demonstrated to express neurotensin receptors that are coupled to Ca^2+^ store release *via* Gq-activated phospholipase C and IP_3_Rs (Borges et al., 1996). Neurotensin itself is expressed by a population of adult chicken ACs that co-express both enkephalin and somatostatin (Watt and Florack, 1994). Interestingly, the levels of all three peptides exhibit light-dependent oscillations in release with the highest level of release occurring in the dark (Yang et al., 1997). Thus, if neurotensin acting on its receptor can link Ca^2+^ elevations with Cl^−^ elevations, we would expect this effect to be most robust under scotopic conditions.

The metabotropic glutamate receptor mGluR5 has also been demonstrated to be linked to the IP_3_ pathway in chick ACs (Sosa et al., 2002; Sosa and Gleason, 2004). In addition, mGluR5 has been localized to ACs in the adult chicken retina at the light microscope level (Kreimborg et al., 2001) and in AC processes in the electron microscope (Sen and Gleason, 2006). Dopamine is a key retinal regulator in the vertebrate retina and D1 (AdC-linked) dopamine receptors are expressed in the inner retina across vertebrates (Veruki and Wässle, 1996; Mora-Ferrer et al., 1999; as reviewed in Nguyen-Legros et al., 1999) including the chicken retina (Firth et al., 1997). Although D1 receptors are linked to elevations in cAMP, it may be that their effect on cytosolic Cl^−^ is relatively small given the limited effects we found for the cAMP cocktail in our experiments. Nonetheless, it remains possible that NO is not the only signaling molecule with the potential to regulate cytosolic Cl^−^. However, given what we have learned about the importance of the Ca^2+^ source, the location of these receptors is likely to be a significant determining factor in their involvement.

Wexler et al. (1998) tested the NO donor SNAP on cultured rat ACs and found that downstream suppression of PKA activity reduced GABA receptor-mediated current. With similar concentrations of SNAP, we have previously demonstrated a small (~15%) enhancement in the GABA-gated current but no shift in E_REV-GABA_ (Hoffpauir et al., 2006). In the present experiments, we see no effect of PKA inhibition on the conductance of the GABA response. In Wexler et al. (1998), PKA activity was modified acutely and effects began to diminish within a few minutes during reagent application and at a rate that would have brought the effect back to baseline in about 10 min. Our PKA inhibitor experiments involved 20 min pre-incubations such that any acute effects could have been reversed by the time we made our recordings. Alternatively, in addition to potential species differences, ACs are a diverse group, and differences in timing and preparation, and maintenance of cultures could easily select for a different sub-population of ACs.

Nitric oxide synthases (NOS) are widespread in the chicken retina (Tekmen-Clark and Gleason, 2013) and there are four subtypes of chick ACs expressed neuronal NOS (nNOS), specifically (Fischer and Stell, 1999). Furthermore, it has been reported that nNOS expression is clustered in the presynaptic region of some ACs dendrites (Cao and Eldred, 2001) and NO imaging shows that diffusion from AC processes is limited (Eldred and Blute, 2005). Together, these observations suggest that although NO can be generated broadly in the retina, its effects could be highly localized to pre-and post-synaptic partners in the inner retina. As we develop a deeper understanding of the signaling mechanisms underlying NO- and CFTR-dependent cytosolic Cl^−^ regulation, we may uncover additional regulators of inhibition that play a role in AC synaptic output and thus the signals that retina ganglion cells convey to visual centers in the brain.

## Data Availability Statement

The raw data supporting the conclusions of this article will be made available by the authors, without undue reservation.

## Ethics Statement

The animal study was reviewed and found to be exempt by the LSU Institutional Animal Care and Use Committee.

## Author Contributions

LZ and EG planned the experiments and drafted the manuscript. LZ carried out all of the experiments. All authors contributed to the article and approved the submitted version.

## Conflict of Interest

The authors declare that the research was conducted in the absence of any commercial or financial relationships that could be construed as a potential conflict of interest.

## Publisher’s Note

All claims expressed in this article are solely those of the authors and do not necessarily represent those of their affiliated organizations, or those of the publisher, the editors and the reviewers. Any product that may be evaluated in this article, or claim that may be made by its manufacturer, is not guaranteed or endorsed by the publisher.
